# Outcomes of Open Subacromial Decompression after Failed Arthroscopic Acromioplasty

**DOI:** 10.5402/2012/806843

**Published:** 2012-05-09

**Authors:** Anand Pillai, Vivek Eranki, Joby Malal, Gavin Nimon

**Affiliations:** ^1^Department of Orthopaedics and Trauma, The Queen Elizabeth Hospital, The University of Adelaide, Woodville, SA 5011, Australia; ^2^Department of Orthopaedics and Trauma, Cairns Base Hospital, Queensland QLD, Australia; ^3^Department of Orthopaedics and Trauma, Dumfries and Galloway Royal Infirmary, Dumfries DG1 4AP, UK

## Abstract

*Aim*. To prospectively assess the effectiveness of revision with open subacromial decompression in patients who had a previous unsatisfactory outcome with the arthroscopic procedure. *Methods*. 11 patients were identified for the study, who did not demonstrate expected improvement in symptoms after arthroscopic acromioplasty. All patients underwent structured rehabilitation. Functional evaluation was conducted using the Hospital for Special Surgery, New York, shoulder rating questionnaire. *Results*. M : F was 7 : 4. The mean age was 57 years. The average shoulder score improved from 49.6 preoperatively to 56 postoperatively at an average followup of 16 months. Two patients showed deterioration in their shoulder scores after revision while the rest showed only marginal improvement. All except one patient stated that they would opt for surgery again if given a second chance. *Conclusion*. In the group of patients that fail to benefit from the arthroscopic decompression, only a marginal improvement was noted after revision with open decompression.

## 1. Introduction

Anterior acromioplasty was first described by Neer in 1972 for chronic impingement syndrome [1]. Since then, this procedure has come to be widely accepted and many surgeons and authors have described consistent and satisfactory results for this treatment [2–6]. Ellman introduced arthroscopic decompression [7] and, in 1991 [8], in a separate study, reported on the two-to five-year results of arthroscopic subacromial decompression which indicated it to be as effective as open decompression as a primary procedure [9–13]. The benefit with arthroscopic repair lies with its recovery time. Mean hospital stay and return to work were found to be significantly better with arthroscopic repair [9, 11, 14]. This facilitated a transition of primary procedure from open procedure to one that is increasingly being performed arthroscopically [1, 2, 8, 9, 15–18]. Arthroscopic acromioplasty has a reported patient satisfaction rate of 67–92% from current literature [8, 9, 11, 12, 15–17].

There is currently no evidence to guide treatment in patients who continue to demonstrate impingement symptoms following arthroscopic decompression.

The aim of this study is to prospectively assess outcomes of revision open subacromial decompression in patients who did not have a satisfactory benefit from a previous arthroscopic acromioplasty.

## 2. Methods

Over a 48-month period, all patients presenting with impingement type symptoms underwent a through clinical examination and MRI. Patients with additional shoulder pathologies and rotator cuff tears were offered treatment but were excluded from the study. All the patients received subacromial injection containing 5 mLs of 1% lidocaine. Patients with impingement symptoms in the absence of other significant shoulder pathologies who responded to diagnostic subacromial injection and were treated with arthroscopic decompression were included in the study. All surgeries were performed by the senior author who is a fellowship trained upper limb surgeon (GN). All patients underwent bursectomy, acromioplasty, and release of the coraco-acromial ligament. Postoperatively all patients underwent a structured rehabilitation and followed up regularly through outpatients. Outcomes were objectively assessed using the L'Insalata shoulder scoring system developed by L'Insalata at the Hospital for Special Surgery, New York which has been validated as a self-administered questionnaire [19]. The self-administered questionnaire consists of five separately scored domains concerning global assessment, pain, daily activities, recreational activities, and work with a higher score indicating a better shoulder function. The original scoring system did not assess patient satisfaction, and therefore separate section for ascertaining the patient's satisfaction with the shoulder was added. The participants were also asked about their willingness to undergo the procedure again and whether they would recommend the procedure to a friend with a similar condition. The questionnaire was administered after the original arthroscopic and the revision open decompression.

After arthroscopic decompression, patients were followed up at 6 weeks, 3 months, 6 months, 9 months, 12 months, and 15 months. Patients who made satisfactory recovery by 15 months were discharged from the clinic and followed up on a “Pro Re Nata” basis. Patients who were unsatisfied with their outcome despite further conservative measures including analgesia and physiotherapy were followed up on a 3 monthly basis and considered for surgery if no improvement was evident. Prior to revision of unsuccessful arthroscopic decompression, patients were once again screened for additional pathologies which could have been missed. Screening was done using clinical evaluation, repeat imaging (MRI), and response to subacromial injection. Only patients who once again displayed greater than 80% relief in pain for greater than 6 hours with subacromial injection containing 5 mLs of 1% lidocaine were considered for revision open decompression.

All patients requiring revision through open decompression were operated by the same senior surgeon (GN). The patient is placed in a beach chair position with the involved upper limb draped free. A sabre cut skin incision is used. The deltoid muscle fibres are split at their anterior and middle third junction, and the muscle flaps are carefully elevated subperiosteally from the anterior acromion. The subacromial space is cleared of any scar tissue or debris. A careful search is made for any offending spurs at the under surface of the acromioclavicular joint, and if found are removed. The subacromial space was assessed in view of need for further acromioplasty and then thoroughly washed out, and the elevated deltoid is reattached to the acromion using absorbable sutures passed through drill holes in the acromion.

Postoperatively the limb is placed in a sling and pendular shoulder movements are encouraged. At two weeks, all shoulder movements are started with self-assistance along with shoulder girdle exercises. By six weeks, the patient graduates to thera-band-resisted internal and external rotation exercises and therapist-assisted abduction exercises. Deltoid strengthening exercises are started at 12 weeks. Postoperatively, the patients were again followed up at 6 weeks, 3 months, 6 months, 9 months, 12 months, and 15 months. At the end of followup, the questionnaire was once again administered to the patients.

## 3. Results

96 patients with isolated impingement symptoms underwent arthroscopic acromioplasty. There were no cases of intra- or postoperative complications with respect to their initial surgery. The patients were followed up in outpatients, and eleven patients were identified who were not satisfied with the outcome of their shoulder function. The mean interval between arthroscopic and revision open procedure was 19 months (range 12–36 months).

The mean age of the study group who underwent open subacromial decompression following arthroscopic acromioplasty was 57 years (range 42–75) and included 7 males and 4 females. Seven patients were operated on the right shoulder while the rest four were on the left. There were no workers compensation claimants in the patient group. The depth of the acromioplasty was found to be appropriate in all cases with no cases requiring further complaining of the acromion. No cuff tears were identified during the revision open decompression.

After revision via open decompression, the patients were followed up for a mean of 16 months (range 12 to 26 months) after the revision surgery. There were no postoperative complications. The shoulder score for the study group improved from a mean of 49.6 (range 28.6 to 89.3) preoperatively to 56 (range 34.3 to 91.3) postoperatively. Two of the patients deteriorated after the revision surgery, while the rest of the nine patients showed only minimal improvement in the shoulder scores. The maximum improvement in the group was by 17.5 points, while the worst performer deteriorated by 11.3 points postoperatively. While the patients in general showed marginal improvement in their global, pain, and daily activity domains, they showed deterioration in their recreation and work domains. Only one patient in the working age group was not at work preoperatively, which deteriorated to 3 patients off work at final followup. The maximal improvement was in the pain domain which improved by a mean of 5.1 points (range 0 to 14). Preoperatively majority of the respondents had rated pain as the domain in which they wanted further improvement which changed to the recreation domain after the revision procedure. Preoperatively 5 patients had rated the satisfaction with their shoulder as poor while the rest 6 rated it as fair. Postoperatively, this improved to 7 patients rating it as good or very good while 4 patients rated it as fair. All except one patient stated that they would opt for surgery again if given a second chance and that they would recommend the surgery to a friend with a similar condition. There was no correlation of patient age, length of followup, or handedness with the final outcome.

## 4. Discussion

Arthroscopic decompression has already been established as an effective first line treatment for impingement syndrome; however, in the small proportion of patients who do not show improvement, there is minimal literature available on revision surgery for unsuccessful acromioplasty [2, 20–22]. Several studies have assessed the success of both open and arthroscopic decompression; however, comparison is difficult due to varying inclusion criteria. In this study, of the 96 patients presented with impingement syndrome and underwent arthroscopic decompression, arthroscopic decompression was successful in 88.5% of cases which is similar to other published series [23, 24]. 11 patients (7 were male and 4 female) had ongoing symptoms and were treated with revision open decompression. The greatest improvement was found to be in the area of pain.

This study would be the first to prospectively assess the outcome of revision open acromioplasty prospectively and has been specifically designed to address the outcome of revision by open repair in patients with impingement symptoms with no other identifiable cause for their shoulder symptoms.

Connor et al. retrospectively reviewed patients' revision of failed anterior acromioplasty in patients with impingement type symptoms and noted greater improvements in pain, function, and recovery rates with arthroscopic decompression compared to open [22]. This has been attributed to arthroscopic surgery being less invasive, allowing earlier and unrestricted postoperative rehabilitation. Studies assessing failure rates of arthroscopic decompression reported rates of approximately 25% via arthroscopic approach and 16% via open approach [23]. The higher failure rates have been attributed to poorer visibility in arthroscopic repair. In the current series, all arthroscopies were satisfactory enough for no further revision to be required. None of the patients required further complaining of the acromion. None of the unsatisfactory results could therefore be attributed to operative technique. To complement the lower failure rates of open decompression, patient satisfaction have been found to be higher with open repair at 94% as opposed to 88% for arthroscopic treatment [11]. The pain relief and function in open decompression has been found to be superior and more predictable compared to arthroscopic decompression [14]. A study by Spangehl et al. found a mean improvement in pain and function of 4.32 for open repair, whereas arthroscopic repair only had an improvement of 2.88 points [12]. A recent study which retrospectively assessed outcomes using the UCLA score after an open revision acromioplasty after failed decompressions for isolated stage II impingement syndrome had 18 good or excellent results out of 35 cases [21]. We had opted for an open acromioplasty as a standard for revision procedures as it was less likely to result in an insufficient decompression and has lower failure rate (16%) [23], greater patient satisfaction, and greater improvement in pain [12, 25]. Another benefit of open repair was also lack of confidence on the part of the patient to undergo a procedure from which they previously did not improve.

Since the failure rates of arthroscopic subacromial decompression surgery can be as high as 10–25% [24], a large number of patients may require subsequent revision surgery. An evidence gap exists for success rates of revision of arthroscopic decompression of shoulders with open decompression in patients' true impingement symptoms. Several studies have examined revision decompression, however, have included patients with multiple significant shoulder pathologies. Hawkins and Abrams explored open anterior decompression following an unsuccessful open anterior acromioplasty [2]. Out of the 51 patients with a failed open anterior acromioplasty, further open decompression was offered to only 11 patients of which only one had a successful outcome as assessed by patient satisfaction and reduction in pain [26]. 65% of their patients were found to have significant pathological causes producing impingement type symptoms such as arthritis and cervical spondylosis. The current series, assessing revision open decompression after arthroscopic decompression, showed superior results with pain and satisfaction with mean pain score improved by a mean of 5.1 points (range 0 to 14). Four patients rated satisfaction as fair and seven as good/very good. Ongoing symptoms or unsatisfactory outcome could be due to chronic tendonitis, subacromial scarring, or the bursa reforming. All patients with detectable shoulder pathologies and rotator cuff tears were excluded. Despite thorough clinical and MRI screening of patients in the current study, intrasubstance defects cannot be detected and could account for less than ideal outcomes after open revision surgery. MRIs of shoulders to visualise the condition of the rotator cuff in patients who have already had arthroscopic decompression could be limited due to metal debris artefact and intrasubstance tears. Several studies assessing revision with open decompression have included patients with significant pathologies and found their outcomes to be significantly inferior [2, 20–22]. In a study by Ogilvie-Harris et al., only 6 out of 11 patients with subacromial adhesions noted improvement in pain from a repeat acromioplasty [20]. Failure of revision surgery has been noted to be frequently associated with conditions that exist in addition to impingement syndrome [21]. Even if both the conditions were successfully treated objectively, the results were not as good as in patients who presented with only impingement [21].

In the absence of significant improvement in the shoulder scores, 7 of the 11 patients were satisfied with their shoulder and all except one stated that they would opt for the surgery again. Patient satisfaction despite less than ideal functional outcome has been observed and reported in previous orthopaedic studies [27, 28]. Satisfaction is affected by many factors unrelated to the surgical intervention. Sernert et al. also found patient expectations to correlate strongest with satisfaction [27]. Given the chronic and debilitating nature of shoulder pain, improvement in function could potentially lead to disproportionate increase in satisfaction. In this case, the high patient satisfaction in the setting of marginal improvement in shoulder function could be attributed to good improvement in pain [11, 14, 28] and pre- op counselling of patients [27] undergoing revision surgery regarding likely outcomes.

Although the number of patients assessed in the study meets the minimum requirement of 9 patients required for validity [19], the low patient numbers in this study is a short coming. Open revision after failed arthroscopic decompression has demonstrated only marginal improvement in scores as per L'Insalata shoulder scale. No statistically significant improvement was noted in the domain of ADLs, recreational activities, and return to work. Pain as a domain demonstrated the greatest improvement with the mean pain score improving by 5.1 points with no patients reporting an increase or worsening in pain. Despite less than ideal outcomes of open revision, 10 of the 11 patients were satisfied with the outcome of the revision.

## 5. Conclusion

Patients fail to show satisfactory improvement after an arthroscopic acromioplasty and are unlikely to benefit from a further open procedure. Prior to undergoing revision by open decompression, patients need to be counselled by their treating surgeons on realistic expectations of post-op function. Though marginal improvement can be expected in shoulder pain, a return to work and recreational activities is unlikely.
